# Errors on Cognitive Testing in Asymptomatic and Prodromal Familial Frontotemporal Dementia and their Prognostic Utility

**DOI:** 10.1002/alz.093114

**Published:** 2025-01-09

**Authors:** Brandon R. Palacios, Mark E. Sanderson‐Cimino, Emily W. Paolillo, Kaitlin B. Casaletto, Jack C. Taylor, Amy B. Wise, Sreya Dhanam, Mai Anh Bui, Hilary W. Heuer, Leah K. Forsberg, Brad F. Boeve, Howard J. Rosen, Adam L. Boxer, Bruce L. Miller, William W. Seeley, Maria Luisa Gorno Tempini, Joel H. Kramer, Adam M. Staffaroni

**Affiliations:** ^1^ Memory and Aging Center, UCSF Weill Institute for Neurosciences, University of California, San Francisco, San Francisco, CA USA; ^2^ Memory and Aging Center, UCSF Weill Institute for Neurosciences, San Francisco, CA USA; ^3^ Department of Neurology, Mayo Clinic, Rochester, MN USA; ^4^ Memory & Aging Center, Department of Neurology, University of California in San Francisco, San Francisco, CA USA; ^5^ Memory and Aging Center, UCSF Weill Institute for Neurosciences, University of California San Francisco, San Francisco, CA USA

## Abstract

**Background:**

The earliest cognitive manifestations of asymptomatic and prodromal familial frontotemporal dementia (f‐FTD) mutation carriers are still being characterized. Patients with symptomatic FTD are known to be error prone, particularly on tasks of executive function, but little is known about the frequency or predictive utility of these errors in the earliest stages of f‐FTD. The current study compared error rates on executive functioning tasks in controls and asymptomatic and prodromal mutation carriers and investigated whether errors predict worsening clinical status as follow up.

**Method:**

This study included 90 participants (mean age = 48.57, SD = 13.30; 51.1% female; 86.67% white) with pathogenic FTD mutations (C9orf72, n = 45; GRN, n = 30; or MAPT, n = 15) who were asymptomatic or prodromal (Clinical Dementia Rating plus FTLD; CDR®+NACC‐FTLD ≤ 0.5). Propensity score matching identified (CDR®+NACC‐FTLD = 0) who did not carry pathogenic mutation but were matched on age, education, and sex. Regressions were used to compare controls and mutation carriers on error scores (rule violations and/or repetitions) from three executive functioning tasks: design fluency, lexical fluency, and Stroop inhibition. Mutation carriers with longitudinal data within 1.5 years were binarized as stable (n = 35) or progressors (n = 13) based on whether their CDR®+NACC‐FTLD sum of boxes score was worse at follow up (mean follow up = 1.07 years). Regressions were fit to evaluate the relationship between baseline error scores and group (i.e., stable vs. progressor).

**Result:**

Mutation carriers committed significantly more errors than controls on the Stroop task (β = 0.67, p = 0.03) and lexical fluency (repetitions: β = 0.27, p = 0.02). The groups did not significantly differ in errors on the remaining tasks. A greater number of Stroop errors predicted mutation carriers who progressed at follow up (β = 1.04, p<0.001). When introducing the primary outcome from each task as a covariate (e.g., correct responses), the fluency task (repetitions: β = 0.10, p = 0.03) and Stroop task (errors: β = 0.82, p<0.001) were significantly associated with progression.

**Conclusion:**

Errors on executive tasks may be early cognitive features in familial FTD. Importantly, errors were uniquely predictive above and beyond total scores. Research in larger samples will enable investigations of genetic differences in error rates.

